# Activity-Weighted Assessment and Environmental Drivers of Compound Ozone–Heat Exposure Risk in Urban Outdoor Exercise Spaces

**DOI:** 10.3390/toxics14070581

**Published:** 2026-06-30

**Authors:** Rui Su, Zhengning Yao, Shuai Zhang, Kailun Zhang, Pengying Du, Lei Yao

**Affiliations:** 1 College of Education, Beijing Sport University, Beijing 100084, China; 2 School of Physical Education and Health, Ningxia Medical University, Yinchuan 750004, China; 3 Key Laboratory of Integration of Sports and Medicine, Ningxia Medical University, Yinchuan 750004, China

**Keywords:** outdoor exercise spaces, compound exposure, ozone pollution, land surface temperature, mobile phone signaling data, activity-weighted exposure, SHAP

## Abstract

Urban outdoor exercise spaces are important public infrastructures for physical activity, but their users may be exposed to concurrent air pollution and unfavorable thermal environmental conditions. This study developed an activity-weighted framework to assess the compound ozone–heat exposure risk in urban outdoor exercise spaces. Taking the central districts of Beijing as the study area, we integrated the mobile phone signaling-derived visitation frequency, 1 km ground-level O_3_ estimates, the 30 m Landsat-derived land surface temperature (LST), the land cover composition, road network indicators, and three-dimensional building morphology variables. An activity-weighted compound ozone–heat exposure risk index (COHER) was constructed by combining the normalized daily visitation frequency, monthly mean O_3_, and area of interest (AOI)-level mean LST. The results showed that the visitation frequency, O_3_, and LST exhibited mismatched spatial patterns, highlighting the need for compound exposure assessment. COHER values ranged from 0.0000 to 0.1918 and were strongly right-skewed, with 49 outdoor exercise spaces identified as the top 10% high-risk sites. These high-risk spaces had a substantially higher visitation frequency and mean LST than the remaining spaces, whereas O_3_ differences were small and not statistically significant. Exploratory XGBoost–SHAP analysis suggested that the built-up intensity, building height variability, and potential airflow obstruction were relatively important environmental correlates of COHER. The proposed framework provides a relative place-based screening tool for identifying priority outdoor exercise spaces for exposure-sensitive planning and risk mitigation.

## 1. Introduction

Urban outdoor exercise spaces are important public infrastructures for promoting physical activity, recreation, and public health. Their value is commonly discussed in terms of accessibility, service coverage, and facility supply [1]. However, these spaces are also environmental exposure settings. During outdoor exercise, users may experience increased inhalation rates and prolonged contact with ambient environmental conditions, making air pollution and heat stress particularly relevant for health-oriented urban space planning [2]. Therefore, evaluating outdoor exercise spaces requires attention not only to whether such spaces are available but also to whether they are environmentally suitable for active use [3,4].

A growing body of international evidence has linked ambient air pollution exposure to adverse cardiovascular and respiratory health outcomes, while outdoor physical activity may increase the relevance of such exposure because of elevated respiration rates and longer contact with ambient air during exercise [2,4]. At the same time, green and blue spaces may provide environmental and health-related buffering effects through pollutant deposition and dispersion, shading, thermal regulation, and psychological restoration [5,6]. These pathways are particularly relevant for outdoor exercise spaces, where the health benefits of physical activity may coexist with potential exposure to air pollution and unfavorable thermal conditions. However, the present study does not aim to estimate observed health outcomes. Instead, it focuses on identifying outdoor exercise spaces where high activity intensity coincides with unfavorable ozone and surface thermal conditions, thereby providing a place-based environmental exposure risk assessment framework.

Ground-level ozone and urban heat represent two important environmental stressors in outdoor activity environments [7]. O_3_ is a secondary photochemical pollutant influenced by precursor emissions, solar radiation, temperature, atmospheric transport, and regional meteorological conditions [8]. Heat exposure, meanwhile, is strongly shaped by the surface thermal properties, land cover, and urban form. These two stressors may overlap in dense urban environments, particularly during periods of strong solar radiation and stagnant atmospheric conditions [9]. For outdoor exercise spaces, such overlap is especially relevant because users are intentionally engaging in physical activity rather than simply passing through these locations. Assessing O_3_ or heat alone may therefore lead to underestimating the practical significance of compound environmental exposure.

Despite the growing interest in urban environmental exposure assessment, several gaps remain. First, many studies evaluate air pollution or heat exposure at the neighborhood, grid, or residential population scale, whereas specific outdoor exercise spaces have received less attention as active exposure settings [10]. Second, exposure assessments often rely on static population or land use indicators, which may not adequately represent the actual use intensity of public activity spaces [11]. Third, air pollution and heat are frequently assessed separately, even though their relevance for outdoor activity may depend on their spatial co-occurrence with human use [12]. These limitations make it difficult to identify outdoor exercise spaces where unfavorable environmental conditions coincide with intensive activity.

Mobile phone signaling data provide an opportunity to address this limitation by approximating the actual visitation frequency at the level of individual outdoor exercise spaces [13]. When combined with gridded air pollution data and satellite-derived thermal information, such data can support a place-based and activity-weighted exposure assessment. In this framework, O_3_ represents the regional photochemical pollution background, the LST characterizes the local surface thermal conditions, and the visitation frequency reflects the population exposure potential of each outdoor exercise space. The surrounding land cover, road networks, and building morphology can further help to interpret the urban environmental context in which a compound exposure risk emerges [14].

Therefore, this study develops an activity-weighted framework for assessing the compound ozone–heat exposure risk in urban outdoor exercise spaces. Taking Beijing as the study area, we integrated the mobile phone signaling-derived visitation frequency, ground-level O_3_ estimates, the Landsat-derived LST, the land cover composition, road network indicators, and three-dimensional building morphology variables [15]. Specifically, this study aimed to (1) characterize the spatial patterns of the visitation frequency, O_3_ exposure background, and LST in outdoor exercise spaces; (2) construct an activity-weighted COHER to identify priority high-risk spaces; and (3) explore the urban environmental correlates of the compound exposure risk using interpretable machine learning. By linking environmental stressors with actual activity intensities, this study provides a place-based perspective for exposure-sensitive planning and risk mitigation in dense urban environments.

## 2. Materials and Methods

### 2.1. Study Area

**Figure 1:**
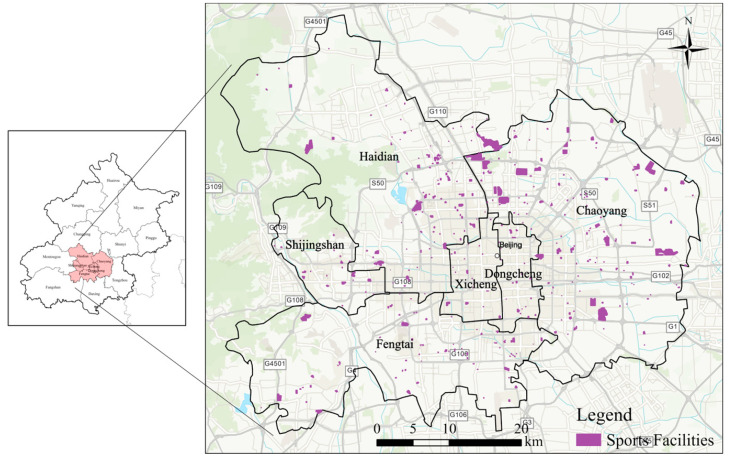
Location of the study area and distribution of sports facilities.

The study focuses on the Dongcheng, Xicheng, Haidian, Fengtai, Shijingshan, and Chaoyang districts of Beijing, China (Figure 1). Beijing is the capital of China and is administratively divided into 16 districts. The six selected districts constitute the main central urban area of Beijing and contain a high concentration of residential communities, employment centers, transportation infrastructure, public services, and recreational facilities.

The study area is characterized by diverse urban functions and heterogeneous built environments. Dongcheng and Xicheng form the historical and administrative core of Beijing, with compact urban fabrics and dense built-up areas. Chaoyang and Haidian are major residential, commercial, educational, and innovation-oriented districts with intensive population mobility and diverse activity spaces. Fengtai and Shijingshan include mixed residential, transport, industrial, and redevelopment areas. These differences in land use, urban morphology, and activity intensity make the area suitable for examining spatial variations in outdoor exercise space visitation.

Beijing has a warm temperate, semi-humid continental monsoon climate, characterized by hot and rainy summers and cold and dry winters. During warm seasons, heterogeneous urban surfaces and built forms can lead to considerable spatial differences in land surface temperature. Therefore, the selected central districts provide a representative urban setting for analyzing the relationship between outdoor exercise space use and surface thermal environments.

### 2.2. Data Sources and Preprocessing

#### 2.2.1. Outdoor Exercise Space Area of Interest (AOI) and Mobile Phone Signaling Data

Outdoor exercise space AOIs were obtained from Baidu Maps and used to define the spatial boundaries of sports and exercise facilities in the study area (Figure 1). The AOI polygons represent the planar extent of each outdoor exercise space and provide the basic spatial units for subsequent environmental exposure assignment and activity intensity characterization. Each AOI was assigned a unique identifier to enable linkage with mobile phone signaling records and other environmental variables. Prior to analysis, the AOI polygons were checked for invalid geometries and projected to appropriate coordinate systems according to the requirements of different spatial operations.

Mobile phone signaling data were used to characterize the actual use intensity of outdoor exercise spaces. The dataset provides anonymized and aggregated visitation records within each exercise space AOI during the study period. The visitation count represents the number of mobile device occurrences detected within the corresponding AOI and was used as a proxy for the intensity of human presence and facility utilization. Because the data were aggregated at the AOI level and did not contain individual trajectories or personally identifiable information, they were suitable for characterizing the place-based activity intensity while preserving user privacy. In this study, the AOI-based mobile phone signaling records were linked to the outdoor exercise space polygons using their unique identifiers.

Because the available mobile phone signaling dataset was aggregated at the AOI level, it was used to derive the average daily visitation frequency, rather than individual dwell times or time-of-day-specific activity durations. DailyVisit was interpreted as a proxy for the place-based activity frequency and population exposure potential. In this study, population exposure potential refers to the relative likelihood that more users may be exposed at a given outdoor exercise space, as approximated by the average daily visitation frequency. It does not represent individual dwell times, physiological exposure doses, or observed health effects.

#### 2.2.2. Ground-Level O_3_ Data

Ground-level ozone (O_3_) concentrations were obtained from the ChinaHighO_3_ dataset, which is part of the ChinaHighAirPollutants (CHAP) product series [16,17]. ChinaHighO_3_ provides seamless, full-coverage, ground-level O_3_ estimates across China at a spatial resolution of 1 km from 2000 to the present, including daily, monthly, and annual products. The daily product represents the maximum daily 8-h average O_3_ concentration (MDA8 O_3_), a commonly used metric for characterizing near-surface ozone exposure.

The dataset was generated by integrating multiple sources of information, including ground-based monitoring observations, satellite remote sensing products, atmospheric reanalysis data, and model simulations, using artificial intelligence methods to capture the spatiotemporal heterogeneity of air pollution. According to the dataset documentation, the daily O_3_ estimates show high predictive performance, with a cross-validation coefficient of determination (CV-R^2^) of 0.89, a root-mean-square error (RMSE) of 15.77 μg m^−3^, and a mean absolute error (MAE) of 10.48 μg m^−3^. In this study, the daily ChinaHighO_3_ D1K products for March 2025 were used to derive the monthly mean near-surface O_3_ exposure background for outdoor exercise spaces.

#### 2.2.3. Land Surface Temperature Data

The land surface temperature (LST) was derived from the Landsat 8 and Landsat 9 Collection 2 Level-2 Surface Temperature products available in Google Earth Engine. These products provide atmospherically corrected surface temperature estimates derived from Landsat thermal infrared observations and are suitable for characterizing fine-scale surface thermal conditions in heterogeneous urban environments. In this study, the ST_B10 band was used and converted to degrees Celsius using the official scale factor and offset:

(1) $$LST{(}^{\circ }C)=DN\times 0.00341802+149.0-273.15$$

All available Landsat 8/9 images covering the study area from 1 March to 31 March 2025 were collected. Fill pixels, dilated clouds, cirrus, clouds, cloud shadows, and snow-contaminated pixels were removed using the QA_PIXEL quality assessment band. The valid LST observations were then composited using the pixel-wise median to generate a 30 m clear-sky monthly representative LST surface for March 2025.

This LST composite represents the relative surface thermal condition under cloud-free Landsat overpass conditions, rather than a continuous daily or monthly mean air temperature. It should be noted that the LST does not directly represent the near-surface air temperature or the physiological thermal conditions experienced by outdoor users. Human thermal conditions and thermal sensation are influenced by multiple meteorological and physiological factors, including the air temperature, relative humidity, solar radiation, wind speed, clothing, metabolic rate, exercise intensity, and exposure duration. Therefore, in this study, the Landsat-derived LST was used only as a proxy for the local surface thermal conditions and fine-scale urban thermal heterogeneity, rather than as a direct measure of human thermal comfort or heat stress.

#### 2.2.4. Land Cover Data

Land cover data were obtained from the China Land Cover Dataset (CLCD), a Landsat-derived annual land cover product for China with a spatial resolution of 30 m [18]. The CLCD provides long-term land cover information for China and includes nine land cover classes: cropland, forest, shrubland, grassland, water, snow/ice, barren land, impervious surface, and wetland. The dataset was developed on the Google Earth Engine platform using Landsat time-series imagery. Temporal metrics derived from available Landsat observations were used as classification features, and a random forest classifier was applied to generate annual land cover maps. Spatial–temporal filtering and logical reasoning were further used to improve the temporal consistency and spatial reliability of the product.

In this study, the CLCD was used to characterize the land cover context surrounding outdoor exercise spaces.

#### 2.2.5. Built Environment Data

Built environment data were used to characterize the urban physical context surrounding outdoor exercise spaces, including the building morphology and road network conditions. Building morphology data were obtained from Amap AOI datasets, including building footprint polygons and floor number attributes. The building footprint data provided the planar extents of individual buildings, while the floor number information was used to estimate vertical building characteristics. Prior to variable extraction, all building polygons were projected to a metric coordinate system, clipped to the study area, and checked for invalid geometries and missing floor number records.

The building height was estimated by multiplying the recorded number of floors by an assumed average floor height of 3 m:

(2) $$BH_{i}=Floor_{i}\times 3$$

where $BH_{i}$ denotes the estimated height of building i, and $Floor_{i}$ represents its recorded number of floors. Based on the processed building footprint and estimated height information, built environment indicators were derived within the surrounding buffers of the outdoor exercise space AOIs, including the building coverage, building density, mean building height, building height variability, building volume, and floor area ratio.

Road network data were obtained from OpenStreetMap (OSM) and used to characterize the traffic-related built environment conditions surrounding outdoor exercise spaces. The road data were projected to the same metric coordinate system and clipped to the study area. Road-related indicators, including the total road density and major road density, were calculated within the surrounding buffers of each AOI. These variables were used to represent the surrounding transport infrastructure and traffic-related urban context, which may be associated with the local air pollution background, surface heat accumulation, and human activity patterns.

### 2.3. Variable Construction and Data Integration

The outdoor exercise space AOI layer was used as the primary spatial framework for integrating all datasets. Each AOI was assigned a unique identifier, which served as the key field for linking AOI-based mobile phone signaling records and extracted environmental variables. Prior to spatial analysis, the AOI polygons were checked for invalid geometries and projected to appropriate coordinate systems according to the requirements of different spatial operations. For vector-based surrounding environmental variables, including road networks and building footprints, the datasets were projected to a common metric coordinate system before buffer-based extraction.

The study period was set to March 2025 because mobile phone signaling records, ChinaHighO_3_ estimates, and valid Landsat-derived LST observations were available and could be matched within the same monthly time window. The purpose of using this period was not to quantify the causal contribution of higher temperatures to O_3_ formation but to demonstrate an activity-weighted, place-based framework for identifying outdoor exercise spaces where the visitation frequency, regional O_3_ exposure background, and local surface thermal conditions co-occur. Therefore, March 2025 was used as a consistent case study period for integrating multi-source spatial datasets.

Data integration was conducted at two spatial levels. First, variables directly representing activity or exposure conditions were assigned at the AOI level. Mobile phone signaling records were linked to each outdoor exercise space AOI using the unique AOI identifier and were used to calculate the average daily visitation frequency. A monthly mean O_3_ was assigned to each AOI based on the 1 km ChinaHighO_3_ grid cell containing the AOI centroid and was interpreted as a regional near-surface ozone exposure background. The mean LST was extracted from the 30 m Landsat monthly composite within each AOI polygon to characterize the local surface thermal conditions.

Second, variables describing the surrounding urban environmental context were extracted within a 500 m buffer around each outdoor exercise space AOI. These variables included the land cover composition, road network indicators, and building morphology indicators. The resulting variables were joined back to the AOI attribute table to form a single AOI-level analytical dataset for COHER calculation, high-risk space identification, and environmental correlation analysis. Detailed geographic information system (GIS) processing steps, variable definitions, extraction procedures, and calculation formulas are provided in Supplementary Methods S1. Because the objective of this study was to identify priority outdoor exercise spaces rather than to compare administrative districts, the outdoor exercise space AOI was retained as the primary analytical unit throughout the analysis. District boundaries were used only to describe the study area and to support the spatial interpretation of the mapped results.

### 2.4. Activity-Weighted Compound Ozone–Heat Exposure Risk Index

To identify outdoor exercise spaces where human activity overlaps with unfavorable atmospheric pollution and thermal environmental conditions, an activity-weighted compound ozone–heat exposure risk index (COHER) was developed. COHER was designed as a relative, place-based screening index rather than a direct epidemiological or clinical health risk metric. The index integrates three process-relevant components: the population exposure potential, derived from the mobile phone signaling-based daily visitation frequency; the regional photochemical pollution background, represented by the near-surface O_3_ concentration; and the local surface thermal conditions, represented by the Landsat-derived LST.

Before index construction, the three variables were normalized using min–max scaling to remove the influence of different units and value ranges:

(3) $$X_{i}^{\prime }=\frac{X_{i}-min(X)}{max(X)-min(X)}$$

where $X_{i}^{\prime }$ denotes the normalized value of variable X for outdoor exercise space i. The normalized variables included the average daily visitation frequency $\left(DailyVisit_{i}^{\prime }\right)$, monthly mean O_3_ concentration $\left(O3_{i}^{\prime }\right)$, and mean LST $\left(LST_{i}^{\prime }\right)$.

A multiplicative formula was adopted because the purpose of COHER was to identify outdoor exercise spaces where high population use coincided spatially with an elevated O_3_ background and higher surface thermal conditions. Under this formula, a high COHER value can occur only when all three components are simultaneously elevated. This differs from an additive formula, in which a very high value in one component could dominate the final score even when the other exposure components are relatively low. Therefore, the multiplicative structure was considered suitable for screening priority spaces characterized by the co-occurrence of a high visitation frequency, higher O_3_ background, and higher surface temperature.

The activity-weighted compound ozone–heat exposure risk index was calculated as:

(4) $$COHER_{i}=DailyVisit_{i}^{\prime }\times O3_{i}^{\prime }\times LST_{i}^{\prime }$$

where $COHER_{i}$ denotes the compound ozone–heat exposure risk of outdoor exercise space i. A higher $COHER_{i}$ value indicates that an outdoor exercise space simultaneously has a higher visitation frequency, a higher O_3_ exposure background, and stronger surface thermal conditions.

The three components were treated symmetrically after normalization because no site-specific epidemiological outcome data, individual exposure durations, or locally calibrated dose–response functions were available to support empirical weighting. Therefore, COHER should be interpreted as a relative, activity-weighted compound exposure screening index rather than a direct measure of physiological doses or observed health risks. Weighted or additive index structures, as well as health-outcome-based approaches, may produce different rankings and should be further examined in future studies when individual-level exposure data and health outcome records become available.

This index was used to support the identification of priority outdoor exercise spaces requiring environmental improvement or risk mitigation. In the subsequent analysis, the land cover composition, road network conditions, and building morphology indicators were further used to interpret the environmental factors associated with spatial variations in compound ozone–heat exposure risk.

### 2.5. Environmental Driver Analysis of Compound Exposure Risk

To further examine the environmental conditions associated with spatial variations in compound ozone–heat exposure risk, an environmental driver analysis was conducted using the land cover, road network, and building morphology variables extracted around the outdoor exercise space AOIs. The activity-weighted compound ozone–heat exposure risk index $\left(COHER_{i}\right)$ was used as the response variable. To avoid circular interpretation, the variables directly used to construct the index, including the average daily visitation frequency, O_3_ concentration, and mean LST, were not included as explanatory variables in this driver analysis. Instead, the model focused on surrounding environmental characteristics that may be associated with the photochemical pollution background, heat accumulation, ventilation conditions, and the environmental buffering capacity.

The explanatory variables included the land cover composition within the 500 m buffer, such as the vegetation proportion, blue space proportion, impervious surface proportion, natural land cover proportion, and other land cover proportions. Built environment variables included road network indicators, such as the total road density and major road density, as well as building morphology indicators, including the building coverage, building density, mean building height, building height variability, building volume, and floor area ratio. These variables were used to characterize the surrounding environmental context of each outdoor exercise space from the perspectives of surface cover, the traffic-related urban intensity, and the three-dimensional built form.

Before modeling, explanatory variables were screened to reduce redundancy. Pairwise correlation analysis was first conducted, and, when two variables showed a strong correlation, the variable with clearer environmental interpretation or stronger relevance to the compound exposure risk was retained. The remaining variables were then used to train an XGBoost regression model using DMSAS version 2.00.1. The model could capture nonlinear relationships and interactions between environmental factors and the compound exposure risk [19,20]. The model can be expressed as:

(5) $$COHER_{i}=f(X_{i})+\varepsilon_{i}$$

where $COHER_{i}$ is the compound ozone–heat exposure risk index of outdoor exercise space i, $X_{i}$ represents the set of surrounding environmental variables, and $\varepsilon_{i}$ is the residual term.

To interpret the fitted model, Shapley Additive Explanations (SHAP) were used to quantify the contribution of each environmental variable to the predicted compound exposure risk. SHAP values provide both global and local interpretations by decomposing model predictions into additive feature contributions:

(6) $${\hat{y}}_{i}=\phi_{0}+\sum_{k=1}^{K}\phi_{i,k}$$

where ${\hat{y}}_{i}$ is the predicted COHER value for outdoor exercise space i, $\phi_{0}$ is the baseline prediction, and $\phi_{i,k}$ denotes the contribution of environmental variable k to the prediction for AOI i. Positive SHAP values indicate that a variable increases the predicted compound exposure risk, whereas negative values indicate a risk-reducing contribution. SHAP summary and dependence plots were used to identify the relative importance, direction, and potential nonlinear thresholds of environmental drivers.

This analysis was not intended to establish causal relationships but rather to provide an interpretable assessment of the environmental conditions associated with an elevated compound ozone–heat exposure risk. The results support the identification of modifiable environmental factors and provide evidence for prioritizing targeted interventions in high-risk outdoor exercise spaces.

## 3. Results

### 3.1. Spatial Distribution of Visitation Frequency, O_3_ Exposure, and LST

**Figure 2:**
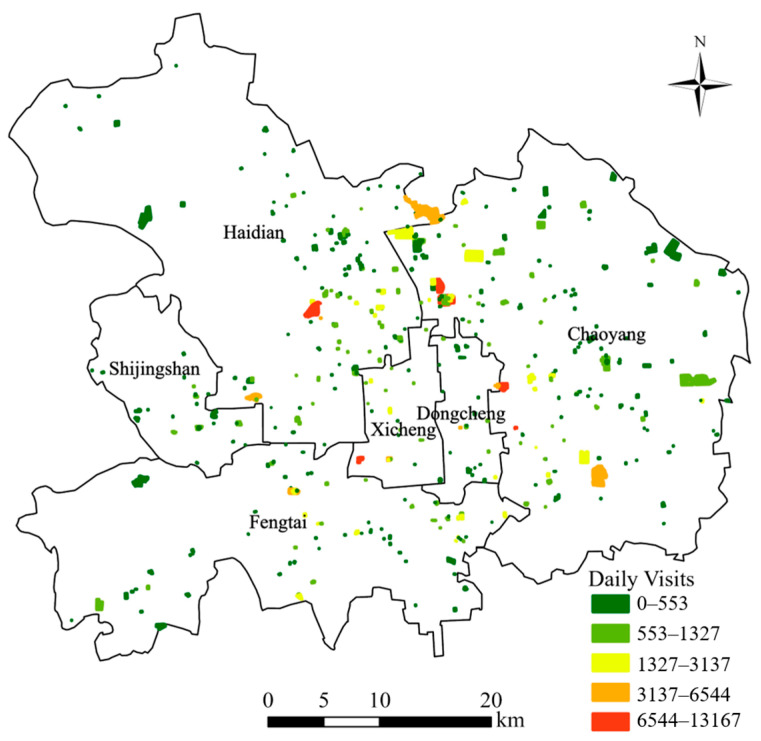
Spatial distribution of average daily visitation frequency in outdoor exercise spaces.

As shown in Figure 2, the average daily visitation frequencies of the outdoor exercise spaces exhibited clear spatial heterogeneity across the study area. Most exercise spaces showed relatively low visitation frequencies, mainly falling within the lowest class of 0–553 visits per day, and were widely distributed across the Haidian, Chaoyang, Fengtai, Shijingshan, Xicheng, and Dongcheng districts. In contrast, high-visitation exercise spaces accounted for a relatively small proportion and displayed a more clustered spatial pattern. These high-value sites were mainly concentrated in the central urban area and several localized clusters, particularly around the boundary areas among Haidian, Xicheng, Dongcheng, and Chaoyang, with a few additional high-visitation sites observed in the southern and eastern parts of the study area. This pattern indicates that potential population exposure, as approximated by the average daily visitation frequency, was unevenly distributed across outdoor exercise spaces, with a limited number of sites carrying substantially higher daily visitation loads than most others.

**Figure 3:**
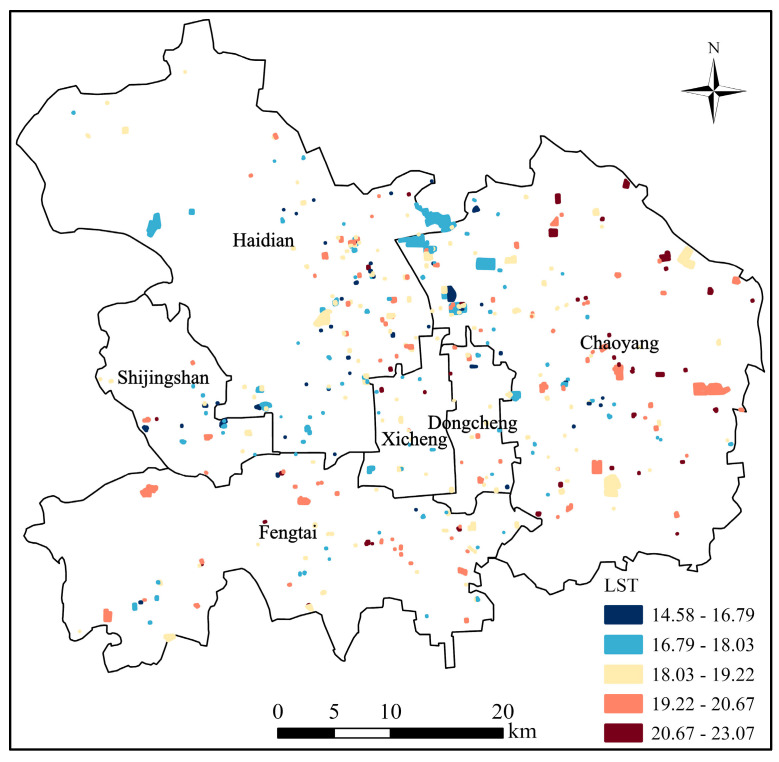
Spatial distribution of mean LST (°C) in outdoor exercise spaces.

The mean LST of the outdoor exercise spaces ranged from 14.58 °C to 23.07 °C and also showed evidence of spatial heterogeneity (Figure 3). Compared with DailyVisit, the LST pattern was more spatially dispersed, with both low- and high-temperature exercise spaces distributed across different districts. Most exercise spaces were concentrated in the intermediate temperature range of 16.79–20.67 °C, while relatively high LST values above 20.67 °C were observed in several localized areas, particularly in parts of Chaoyang, Fengtai, Dongcheng, and the eastern urban area. Low-LST exercise spaces were scattered across the study area and were more frequently observed in locations with potentially better vegetation coverage, open space conditions, or a lower surrounding built-up intensity. In contrast, high-LST exercise spaces were also observed in several highly urbanized areas. The latter pattern was consistent with the environmental comparison presented below, but it should be interpreted as descriptive spatial evidence rather than as direct evidence.

**Figure 4:**
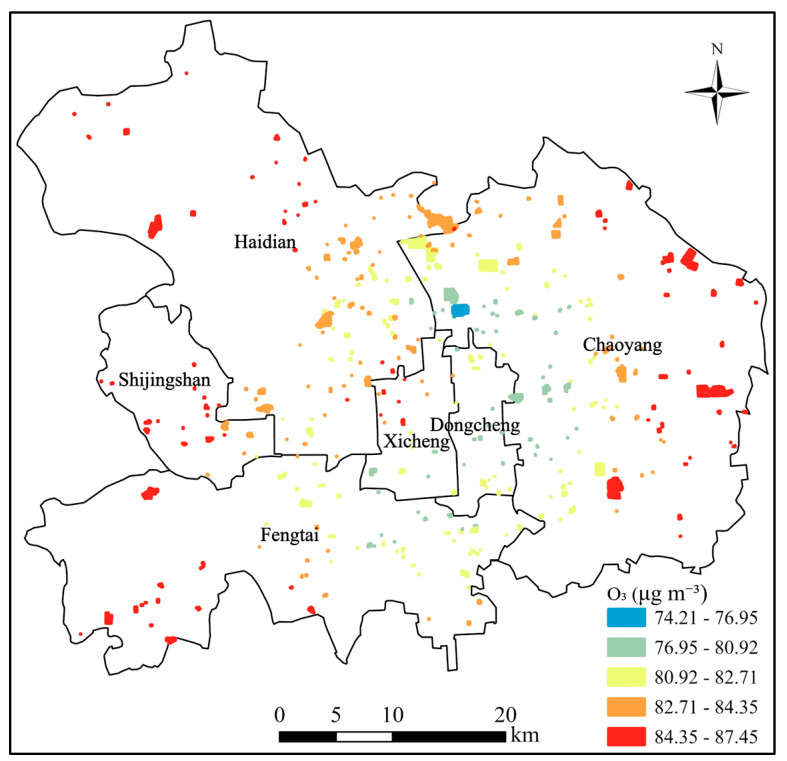
Spatial distribution of monthly mean O_3_ concentration (μg m^−3^) in outdoor exercise spaces.

The monthly mean O_3_ concentration assigned to the outdoor exercise spaces ranged from 74.21 to 87.45 μg m^−3^ and exhibited a clear regional gradient (Figure 4). Compared with the visitation frequency and LST, the O_3_ pattern showed a smoother regional gradient because the O_3_ data had a 1 km spatial resolution and were assigned to exercise spaces based on the grid cell containing each AOI centroid. Therefore, O_3_ was interpreted as a regional near-surface exposure background rather than a site-specific intra-AOI concentration. Lower O_3_ values were mainly observed in the central urban area, particularly around Xicheng, Dongcheng, and adjacent areas. In contrast, higher O_3_ values were more frequently distributed in peripheral areas, including Northern and Western Haidian, Shijingshan, Southern Fengtai, and Eastern and Northeastern Chaoyang. This mismatch among the spatial patterns of O_3_, the LST, and the visitation frequency suggests that single-factor exposure assessment may fail to capture the compound risk faced by outdoor exercise spaces. Therefore, integrating the O_3_ exposure background with the local thermal conditions and activity intensity is necessary for identifying locations where active population use coincides with unfavorable atmospheric and thermal environments.

### 3.2. Compound Ozone–Heat Exposure Risk

This section presents the distribution of the activity-weighted COHER index and identifies priority outdoor exercise spaces with a higher compound exposure risk. Because the thermal component was derived from the Landsat LST, the results should be interpreted as reflecting the surface thermal conditions rather than the continuous air temperature.

#### 3.2.1. Distribution and Spatial Pattern of COHER

**Figure 5:**
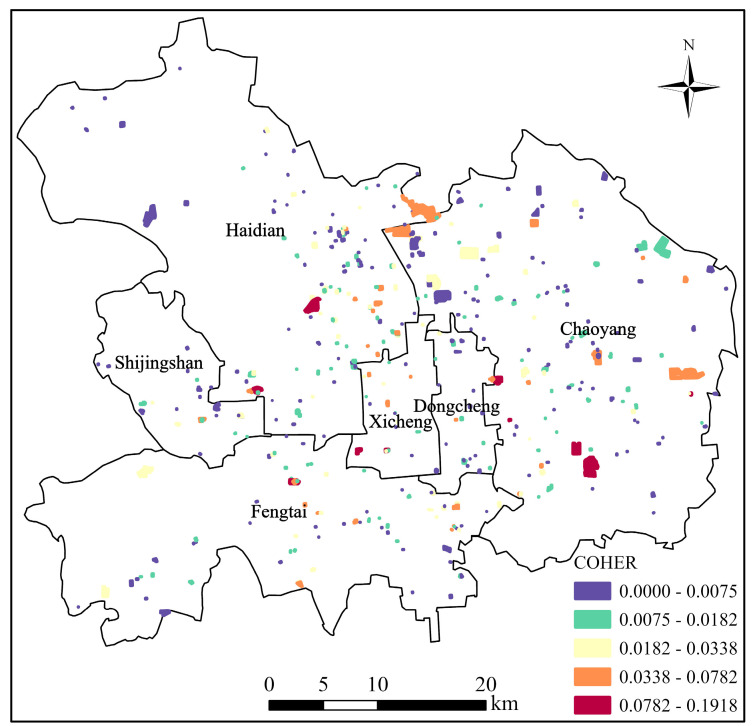
Spatial distribution of the COHER index across outdoor exercise space AOIs.

As shown in Figure 5, the activity-weighted COHER exhibited pronounced spatial heterogeneity across outdoor exercise spaces. The COHER values ranged from 0.0000 to 0.1918, with a mean value of 0.0143 and a median value of 0.0072, indicating a strongly right-skewed distribution. The 75th and 90th percentile values were 0.0176 and 0.0325, respectively, suggesting that most outdoor exercise spaces had a relatively low compound exposure risk, whereas a small subset showed substantially higher risk levels.

The spatial pattern of COHER was not identical to that of any single component variable but reflected the combined influence of the visitation frequency, O_3_ exposure background, and LST. Lower-COHER spaces were widely distributed across the study area, particularly in locations where at least one of the three components remained low. In contrast, higher-COHER spaces were relatively sparse and displayed localized clustering. Several higher-risk sites were observed around the central urban area and its surrounding districts, especially near the boundary areas among Haidian, Xicheng, Dongcheng, and Chaoyang. Additional higher-COHER sites were also identified in parts of Eastern Chaoyang and southern urban areas.

Based on the top 10% threshold of COHER, 49 outdoor exercise spaces were identified as priority high-risk sites. These locations represent areas where a high visitation frequency coincides with relatively unfavorable atmospheric and surface thermal conditions. To further characterize these priority sites, the component variables of spaces in the top 10% of the COHER distribution were compared with those of the remaining spaces (Table 1). According to the Mann–Whitney U test, spaces in the top 10% of the COHER distribution had significantly higher DailyVisit and mean LST values than the remaining spaces (both *p* values < 0.001). Specifically, the mean DailyVisit of the top 10% sites was approximately 2764 visits per day, more than five times that of the remaining sites, while their mean LST was about 0.96 °C higher. The mean LST difference between the two groups was statistically significant but modest in magnitude; therefore, it should be interpreted as part of the relative COHER ranking rather than direct evidence of perceived thermal discomfort or health risk. In contrast, the difference in the monthly mean O_3_ concentration between the two groups was small and not statistically significant (*p* = 0.704). As expected, COHER was significantly higher in the top 10% group because this group was defined using the top 10% COHER threshold. These results indicate that higher-COHER spaces were mainly distinguished by higher visitation frequencies and elevated local surface thermal conditions under broadly comparable regional O_3_ backgrounds. This also indicates that, for the March 2025 case study period, O_3_ contributed to COHER as a regional background exposure component, but it contributed less to differentiating the top 10% high-COHER spaces from the remaining spaces than the visitation frequency and LST.

Overall, the right-skewed distribution and localized concentration of the top 10% group indicate that the combined exposure burden was unevenly distributed across outdoor exercise spaces. Instead, a small subset of highly used and environmentally stressed sites contributed disproportionately to the overall place-based compound exposure burden. This highlights the importance of integrating human activity, the air pollution background, and thermal conditions when identifying outdoor spaces requiring priority environmental improvement or risk mitigation.

#### 3.2.2. Identification and Characteristics of High-Risk Spaces

Beyond the component variables used to construct COHER, the top 10% high-COHER spaces also showed statistically significant differences in several surrounding environmental characteristics. According to the Mann–Whitney U test, higher-COHER spaces had a significantly higher impervious surface proportion than the remaining spaces (0.939 vs. 0.898, *p* = 0.021), while their other land cover proportions were significantly lower (0.058 vs. 0.093, *p* = 0.016). In contrast, the vegetation proportion, blue space proportion, and natural land cover proportion did not differ significantly between the two groups.

For the building morphology, the top 10% high-COHER spaces showed a significantly higher building coverage ratio (BCR; 0.219 vs. 0.171, *p* < 0.001), building volume density (BVD; 2.96 vs. 2.36, *p* = 0.015), floor area ratio (FAR; 0.986 vs. 0.787, *p* = 0.015), and frontal area index (FAI; 0.137 vs. 0.110, *p* = 0.012). However, differences in the mean building height, building height standard deviation, compactness index, total road density, and major road density were not statistically significant. These results suggest that priority higher-COHER spaces were mainly associated with higher imperviousness and more intensive surrounding built forms, rather than with statistically significant differences in road density or green–blue space proportions.

### 3.3. Interpretable Analysis of Environmental Correlates

#### 3.3.1. Relative Importance of Environmental Variables

To further explore the environmental characteristics associated with the compound ozone–heat exposure risk, an XGBoost model was fitted using COHER as the response variable and the surrounding land cover, road network, and building morphology indicators as predictors. Variables directly used to construct COHER, including DailyVisit, the O_3_ concentration, and the LST, were excluded from the predictor set to avoid circular interpretation. The model showed limited predictive performance, with a test set R^2^ of 0.0374, RMSE of 0.0230, and MAE of 0.0135. Given the low test set R^2^, the SHAP results were interpreted only as exploratory indications of relative environmental associations rather than strong predictive relationships, definitive drivers, or causal effects.

**Figure 6:**
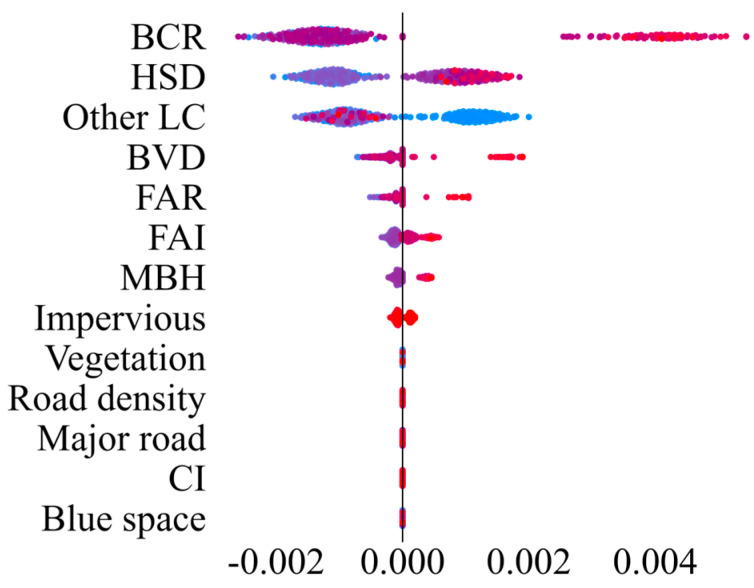
SHAP summary plot showing the distribution of feature contributions. Point colors represent the relative feature values, with blue indicating lower values and red indicating higher values. Note: SHAP, Shapley Additive Explanations; BCR, building coverage ratio; HSD, building height standard deviation; BVD, building volume density; FAR, floor area ratio; FAI, frontal area index; MBH, mean building height; CI, compactness index.

As shown in Figure 6, building morphology variables contributed most prominently to the model interpretation. The building coverage ratio (BCR) had the highest mean absolute SHAP value, indicating that the horizontal intensity of surrounding built-up areas was the most informative environmental variable in the exploratory model. Building height variability (HSD) and other land cover proportions also ranked among the most important variables, followed by the building volume density (BVD), floor area ratio (FAR), frontal area index (FAI), mean building height (MBH), and impervious surface proportion. In contrast, the vegetation proportion, road density, major road density, compactness index (CI), and blue space proportion showed relatively small SHAP contributions.

The SHAP beeswarm plot further suggested that the relationship between the environmental variables and predicted COHER was heterogeneous and nonlinear (Figure 6). Higher BCR values generally contributed positively to the predicted COHER, whereas lower BCR values were mostly associated with negative SHAP values. Similar positive contributions were observed for higher BVD, FAR, FAI, MBH, and HSD values, indicating that exercise spaces embedded in more intensive and vertically complex built environments tended to be associated with a higher predicted compound exposure risk. By contrast, other land cover proportions showed the opposite pattern, with higher values generally contributing negatively to the predicted COHER. These results suggest that, within the exploratory model, the built-up intensity and vertical urban morphology were more strongly associated with COHER variations than road network indicators or blue space conditions.

Overall, because the environmental variables explained only a limited proportion of the total variation in COHER, the SHAP results should be interpreted cautiously. The analysis provides exploratory information about the relative ranking and direction of environmental associations, but this should not be interpreted as evidence that these variables strongly predict or cause higher COHER. Within this limited exploratory model, built-form indicators appear more informative than road network indicators or the blue space proportion.

#### 3.3.2. Nonlinear Associations of Key Environmental Factors

**Figure 7:**
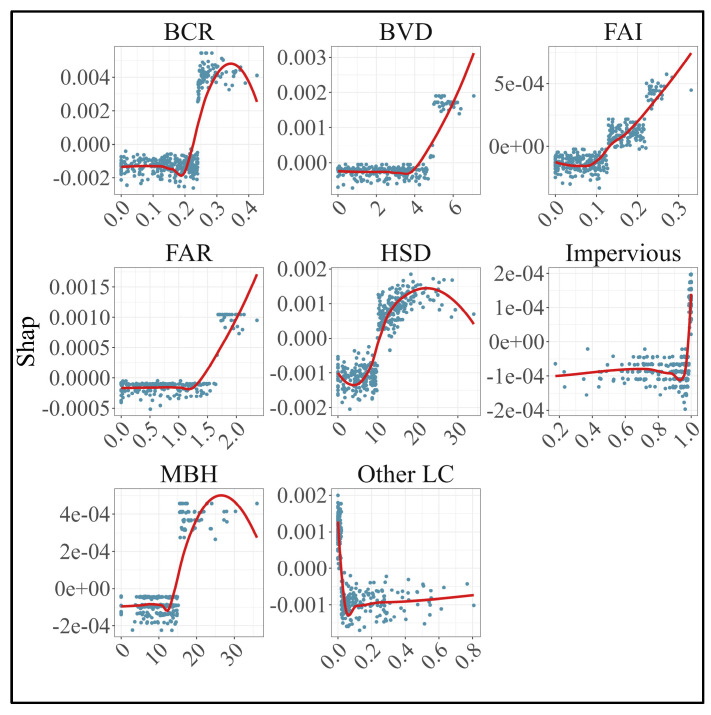
SHAP dependence plots for key environmental variables associated with predicted COHER. Blue dots represent individual observations, and the red line represents the fitted trend showing the overall relationship between the feature value and the SHAP value. Note: SHAP, Shapley Additive Explanations; BCR, building coverage ratio; BVD, building volume density; FAR, floor area ratio; FAI, frontal area index; HSD, building height standard deviation; MBH, mean building height.

The SHAP dependence plots were used to provide an exploratory visualization of potential nonlinear associations between selected environmental variables and the predicted COHER (Figure 7). Given the low test set R^2^ of the XGBoost model, these dependence plots were interpreted as providing broad indicative patterns rather than precise thresholds or strong predictive relationships.

Within this exploratory framework, several built form indicators, including BCR, BVD, FAR, FAI, HSD, and MBH, generally showed positive SHAP patterns at higher values, suggesting that more intensive and vertically complex surrounding built environments may be relatively associated with a higher predicted COHER. The impervious surface proportion also showed a weak positive pattern at very high values, whereas other land cover proportions generally showed a negative SHAP pattern. However, these patterns should be interpreted cautiously because the model explained only a small proportion of the COHER variation.

Overall, the dependence plots suggest that the environmental associations with COHER may be nonlinear, but they do not provide sufficient evidence to define precise threshold values or to establish strong environmental correlates. Therefore, the SHAP results are used here only to complement the group comparison results and to identify potential environmental patterns for future investigation.

## 4. Discussion

### 4.1. Added Value of Activity-Weighted Compound Exposure Assessment

Traditional assessments of environmental exposure in urban outdoor spaces often focus on either pollutant concentrations or thermal conditions, while the intensity of human use is less explicitly considered [21]. However, for outdoor exercise spaces, environmental risk is not determined solely by whether a place has a high O_3_ concentration or high surface temperature. It also depends on whether these unfavorable environmental conditions coincide with active population use. In this regard, the activity-weighted COHER developed in this study provides a place-based perspective for identifying outdoor exercise spaces where environmental stress and human activity overlap.

The spatial patterns observed in this study demonstrate the necessity of such an integrated assessment. The daily visitation frequency, O_3_ exposure background, and LST showed different spatial structures [22]. High-visitation sites were concentrated in several central and localized clusters, whereas O_3_ exhibited a clearer regional gradient, with higher values more frequently observed in peripheral areas. The LST showed more dispersed local variation across different districts. These mismatched spatial patterns indicate that a single-factor assessment may lead to misclassifying priority spaces [23,24]. For example, a site with high O_3_ concentrations but low visitation may be less relevant from a population exposure perspective than a site where moderate-to-high O_3_ concentrations and an elevated LST coincide with a high visitation frequency. Similarly, a thermally stressed site may not necessarily constitute a priority for intervention if it is rarely used [25].

The COHER results further support this argument. Most outdoor exercise spaces showed relatively low compound exposure risks, while a small subset of sites had much higher COHER values. The top 10% high-COHER spaces were characterized by substantially higher average daily visitation frequencies and moderately higher LSTs than the remaining spaces, whereas their mean O_3_ concentrations differed only slightly [10,26]. This suggests that a high compound exposure risk is not simply a reflection of the highest pollutant concentrations or highest thermal values but rather the spatial co-occurrence of environmental stressors with intensive outdoor activity [27]. Therefore, activity weighting changes the interpretation of environmental exposure from a purely concentration-based assessment to a population-relevant exposure perspective.

The limited difference in O_3_ concentrations between the top 10% high-COHER spaces and the remaining spaces has important implications for interpreting the index. In the March 2025 case study period, higher-COHER spaces were mainly distinguished by substantially higher visitation frequencies and moderately higher LSTs, whereas the O_3_ difference between the two groups was small and not statistically significant. Therefore, the higher-COHER classification should not be interpreted as indicating that these spaces had markedly higher ozone concentrations than other spaces. Rather, it indicates that higher human use and elevated local surface thermal conditions occurred under broadly comparable regional O_3_ backgrounds.

This pattern is partly related to the spatial scale and variability of the O_3_ data. The ChinaHighO_3_ product used in this study has a 1 km spatial resolution, and O_3_ values were assigned to outdoor exercise spaces based on the grid cell containing each AOI centroid. As a result, O_3_ was represented as a regional near-surface exposure background rather than a fine-scale site-level concentration. Under such conditions, the O_3_ component functions more as a background pollution component in the COHER framework, while the visitation frequency and LST contribute more strongly to spatial differentiation among outdoor exercise spaces. This reinforces the need to interpret COHER as a relative place-based screening index, rather than as evidence that air pollution is the dominant source of variation in all identified high-risk spaces.

This distinction is particularly important for outdoor exercise spaces because these places are intentionally used for physical activity. Exercise increases respiration rates and time spent outdoors, which may increase the relevance of ambient air pollution and heat exposure for users [28]. Although this study did not estimate individual physiological doses, incorporating the mobile phone signaling-derived visitation frequency allows the assessment to better approximate where environmental conditions are more likely to affect larger active populations. Thus, the proposed framework can help to shift urban environmental assessment from identifying “polluted” or “hot” places alone to identifying “high-use places under unfavorable environmental conditions” [29].

### 4.2. Urban Environmental Correlates of Compound Exposure Risk

The results suggest that the compound ozone–heat exposure risk is not only shaped by the direct components of the COHER index but is also associated with the broader urban environmental context surrounding outdoor exercise spaces [30]. It should be noted that the high-risk-space comparison and the XGBoost–SHAP analysis addressed two related but distinct questions. The former compared the characteristics of the top 10% high-COHER spaces with those of the remaining spaces, whereas the latter examined environmental correlates of COHER across all outdoor exercise spaces. Therefore, the following discussion interprets the high-risk group comparison as evidence of the environmental profile of priority spaces, while the SHAP results are interpreted as exploratory evidence of relative environmental associations across the full sample.

The comparison between the top 10% high-COHER spaces and the remaining spaces showed that priority spaces were associated with several statistically significant features of more urbanized surroundings. Specifically, higher-COHER spaces had significantly higher impervious surface proportions and significantly lower other land cover proportions. In terms of building morphology, they showed significantly higher BCR, BVD, FAR, and FAI. In contrast, the vegetation proportion, blue space proportion, natural land cover proportion, road density indicators, MBH, HSD, and CI did not show statistically significant differences between the two groups. Therefore, the high-risk-space profile should be interpreted mainly as higher imperviousness and a more intensive surrounding built form, rather than as a general deficiency in green–blue space or a broad increase in all building morphology indicators. Although green and blue spaces are widely recognized as potential environmental buffers, their proportions did not show statistically significant differences between higher-COHER spaces and the remaining spaces in the present study [31]. The statistically supported patterns of higher imperviousness and a more intensive built form are consistent with previous studies showing that impervious surfaces and a dense urban morphology can contribute to heat storage, reduced openness, and weaker near-surface ventilation, thereby creating less favorable outdoor environmental conditions under ozone–heat stress [7].

The full-sample SHAP analysis provided a complementary perspective. Although the XGBoost model based only on surrounding environmental variables showed limited predictive performance, the relative importance ranking consistently highlighted building morphology variables [32]. The building coverage ratio had the highest SHAP contribution, followed by building height variability, other land cover proportions, the building volume density, the floor area ratio, the frontal area index, the mean building height, and the impervious surface proportion [33]. This pattern suggests that, across all outdoor exercise spaces, the spatial variation in COHER was more closely associated with the built-up intensity and vertical urban form than with road network indicators or sparse green–blue space variables. In other words, the environmental correlates of the compound exposure risk were more strongly expressed through the physical configuration of the surrounding built environment than through the road density alone [34].

The nonlinear SHAP dependence patterns further indicate that the association between urban form and COHER is not simply linear. The building coverage ratio, building volume density, floor area ratio, frontal area index, mean building height, and building height variability showed threshold-like positive associations with the predicted COHER. Their SHAP values tended to shift from negative or near-zero contributions to positive contributions after reaching moderate levels [35]. This suggests that the influence of the built-up form may become more evident once the building coverage, volume, development intensity, or airflow obstruction exceed certain levels. Such nonlinear patterns are consistent with the idea that a dense urban morphology may affect the compound exposure risk through multiple pathways, including increased surface heat storage, reduced sky openness, and weakened ventilation efficiency [36].

Land cover variables showed a more nuanced pattern. High-risk spaces had higher impervious surface proportions and lower natural land cover proportions than other spaces, supporting the interpretation that highly urbanized surroundings are associated with an elevated compound exposure risk [37,38]. However, the vegetation and blue space proportions had relatively small SHAP contributions in the full-sample model. This should not be interpreted as evidence that vegetation or blue space is unimportant. Rather, it likely reflects the data structure of the present study: most exercise space buffers contained very limited vegetation and blue space coverage, resulting in highly zero-inflated distributions and limited variation for the model to exploit. The weak SHAP contribution of green–blue variables therefore suggests that their buffering role may be difficult to detect when such elements are sparse or unevenly distributed around outdoor exercise spaces [39].

Road network indicators also showed limited associations with COHER. The total road density and major road density did not emerge as important SHAP variables, and the high-risk group did not show a markedly higher road density than other spaces. One possible explanation is that the O_3_ variable used in this study represents a regional photochemical pollution background rather than near-road primary traffic pollution [40]. O_3_ is a secondary pollutant influenced by photochemical formation, atmospheric transport, titration processes, and meteorological conditions [41]. Therefore, the local road density may not correspond directly to higher O_3_ exposure at the scale of outdoor exercise spaces. This result also suggests that, in the current framework, the compound exposure risk is more strongly differentiated by the activity intensity, thermal conditions, and built-environment morphology than by the road network density.

Overall, the urban environmental correlates identified in this study point to the importance of the built-environment configuration in shaping the compound ozone–heat exposure risk. High-risk spaces were generally characterized by a high visitation frequency, higher local surface thermal conditions, greater imperviousness, a stronger built-up intensity, and higher potential airflow obstruction. Meanwhile, the limited predictive performance of the XGBoost model indicates that these environmental variables should not be interpreted as deterministic drivers of COHER. Instead, they provide exploratory evidence of the environmental contexts in which an elevated compound exposure risk is more likely to occur. This distinction is important for translating the results into planning practice: mitigation should not rely on a single environmental variable but should consider the combined configuration of land cover, the building morphology, the ventilation potential, and the actual human use intensity [42].

### 4.3. Implications for Planning and Risk Mitigation

The activity-weighted compound exposure perspective provides a practical basis for prioritizing environmental management in urban outdoor exercise spaces [5]. The results showed that higher-COHER spaces were not simply locations with the highest O_3_ concentration or the highest LST, but places where relatively unfavorable atmospheric and thermal conditions overlapped with intensive human use. This distinction is important for planning practice because environmental interventions are often constrained by limited land, budgets, and management capacities [43]. Rather than treating all polluted or hot spaces equally, the COHER framework helps to identify outdoor exercise spaces where environmental stress is more likely to coincide with large active populations and therefore where interventions may be of greater public health relevance.

For higher-COHER spaces, heat mitigation should be a primary planning consideration. The top 10% high-risk spaces had a higher mean LST than other spaces, and the SHAP results further indicated that a higher built-up intensity and airflow obstruction were associated with an increased predicted COHER. These findings suggest that the local thermal conditions in outdoor exercise spaces may be amplified by a compact surrounding urban form and limited ventilation potential [44]. Planning measures such as increasing tree canopy, providing continuous shading along activity areas, using high-albedo or permeable surface materials, and reducing large expanses of exposed impervious surfaces can help to mitigate surface heat stress [45]. In high-use exercise spaces, these interventions should be placed not only for esthetic improvement but also for reducing exposure during periods when outdoor physical activity overlaps with elevated thermal conditions.

The results also highlight the need to strengthen environmental buffering around outdoor exercise spaces. High-risk spaces were characterized by higher impervious surface proportions and lower natural land cover proportions, whereas vegetation and blue space variables showed weak model contributions, partly because they were sparse in most buffers. This suggests that many exercise spaces lack sufficient green–blue infrastructure to provide effective local cooling or buffering. In dense urban areas where large new green spaces are difficult to create, small-scale interventions may still be meaningful, including increasing roadside vegetation, adding shade trees around sports fields, improving the planting continuity along access routes, and incorporating water-sensitive or cooling landscape elements where feasible [46]. Such measures may be especially important for heavily used exercise spaces embedded in highly impervious surroundings.

The urban morphology should also be considered in exposure-sensitive planning. The positive associations of BCR, BVD, FAR, MBH, HSD, and FAI with the predicted COHER suggest that compact and vertically complex built environments may be linked to higher compound exposure risks [47]. For existing high-risk spaces, mitigation should focus on preserving ventilation corridors, avoiding additional obstruction around activity areas, and improving the openness of surrounding public spaces. For newly planned exercise spaces, site selection should consider not only accessibility and service coverage but also the surrounding building density, vertical form, and potential for airflow blockage [48]. Locating new outdoor exercise spaces in areas with better openness, lower imperviousness, and stronger green–blue buffering may reduce the likelihood of future compound exposure risks.

In addition to physical planning, time-sensitive risk management is necessary. O_3_ pollution and heat stress are both temporally dynamic and may occur concurrently under unfavorable meteorological conditions, thereby increasing the potential health risks during outdoor activities [49]. Outdoor exercise behavior also varies by time of day. Although this study used monthly aggregated exposure indicators, the identification of high-use and high-risk spaces can support more targeted operational measures. For example, higher-COHER spaces could be prioritized for public risk communication during high-O_3_ or high-temperature episodes, including advisories to avoid intense outdoor exercise during unfavorable periods, the provision of shaded rest areas, and the flexible scheduling of organized sports activities [50]. Such measures are particularly relevant for populations who frequently use outdoor exercise spaces and may experience elevated inhalation rates during physical activity.

Overall, the findings support a shift from supply-oriented outdoor exercise space planning toward exposure-sensitive management. Traditional planning often emphasizes the quantity, accessibility, and service coverage of sports and recreational spaces. While these dimensions remain important, the present results suggest that environmental exposure conditions should also be incorporated into planning and management decisions. An effective strategy should jointly consider who uses the space, when it is used, and what environmental conditions users encounter [51]. By integrating the mobile phone signaling-derived activity intensity with air pollution and thermal indicators, the proposed framework can help urban managers to identify priority spaces for targeted mitigation, optimize resource allocation, and promote healthier outdoor physical activity environments in dense urban settings.

### 4.4. Limitations and Future Research

Several limitations should be acknowledged. First, the O_3_ data used in this study represent the regional near-surface ozone exposure background at a spatial resolution of 1 km. Because most outdoor exercise space AOIs are much smaller than the O_3_ grid cell, the assigned O_3_ values should be interpreted as a regional photochemical pollution background rather than fine-scale intra-site ozone concentrations. This scale mismatch may obscure local variations caused by the street-level morphology, vegetation, micro-scale ventilation, and short-term atmospheric processes [15]. Future studies could integrate higher-resolution air pollution simulations, ground-based monitoring, or low-cost sensor observations to better capture the fine-scale ozone variability around outdoor exercise spaces. In addition, because the O_3_ concentrations varied only modestly across the outdoor exercise spaces during the March 2025 case study period, the O_3_ component had a limited ability to distinguish the top 10% high-COHER spaces from the remaining spaces. Therefore, the current COHER results should be interpreted as identifying high-use and warmer outdoor exercise spaces under a broadly comparable regional O_3_ background, rather than locations with distinctly higher ozone pollution.

Second, the LST was derived from Landsat clear-sky observations and represents the surface thermal conditions under satellite overpass conditions. Although the LST is useful for characterizing spatial differences in surface thermal conditions, it does not directly represent the air temperature, perceived heat stress, or physiological heat exposure experienced by users. Human thermal comfort is also affected by humidity, wind speeds, radiation, shading, clothing, and activity intensities [52]. Future research should incorporate the air temperature, humidity, wind, solar radiation, and human–biometeorological indicators such as UTCI or WBGT to more comprehensively characterize outdoor heat exposure during physical activity [53].

Third, the mobile phone signaling data used in this study were aggregated at the AOI level and did not include individual dwell times, repeated stay durations, or time-of-day-specific activity information. Therefore, DailyVisit represents the average daily visitation frequency rather than the actual duration of exposure experienced by individual users. This limitation means that COHER should be interpreted as a visitation frequency-weighted, place-based exposure risk indicator, rather than an exposure dose indicator based on individual residence times. Future studies could use higher-temporal-resolution mobile phone signaling data to estimate dwell times within outdoor exercise spaces and stratify activity durations by time of day, such as morning, afternoon, and evening periods [54].

Fourth, this study was based on a single-month case study period. March 2025 was selected because the mobile phone signaling data, ChinaHighO_3_ estimates, and valid Landsat LST observations could be integrated within the same time window. However, March does not represent peak summer ozone–heat conditions in Beijing, and a single-month observation window cannot capture the seasonal or interannual variability of O_3_ concentrations, surface thermal conditions, or outdoor exercise behavior. Therefore, the results should be interpreted as reflecting a one-month case study assessment rather than as the full-year, peak-summer, or temporally stable characterization of the compound ozone–heat exposure risk. Future studies should extend the framework to warmer and higher-O_3_ periods, such as July and September, or to multi-season datasets, to examine whether the spatial ranking of high-risk outdoor exercise spaces remains consistent across different meteorological and activity conditions [55]. Another important limitation concerns the use of the Landsat-derived LST as the thermal component of COHER. Although the LST is useful for capturing fine-scale surface thermal heterogeneity in urban environments, it does not directly correspond to the near-surface air temperature or to the thermal stress experienced by outdoor users. Human thermal sensation is jointly shaped by the air temperature, humidity, radiation, wind speed, clothing, metabolic rate, exercise intensity, and exposure duration. Therefore, the LST component of COHER should be interpreted as an indicator of the relative surface thermal conditions rather than a direct measure of human thermal comfort or physiological heat stress. Future studies should incorporate the air temperature, humidity, wind speed, radiation, and biometeorological indices such as UTCI, PET, or WBGT to better evaluate human thermal exposure in outdoor exercise spaces.

Fifth, the environmental driver analysis was exploratory. To avoid circular interpretation, variables directly used to construct COHER, including DailyVisit, O_3_, and LST, were excluded from the XGBoost predictor set. As a result, the model based on surrounding environmental variables showed limited predictive performance. The SHAP results should therefore be interpreted as relative environmental associations rather than causal mechanisms or strong predictive controls. Future studies could improve the explanatory framework by incorporating additional process-related variables, such as the sky view factor, urban canyon geometry, local wind fields, anthropogenic heat, shading conditions, population accessibility, and facility characteristics [56].

Sixth, this study did not include health outcome data, such as outpatient asthma visits or other cardiovascular–respiratory indicators. Therefore, COHER should be interpreted as a place-based environmental exposure risk indicator rather than direct evidence of observed health effects. Future studies could link COHER with age-stratified health outcome data, such as outpatient asthma visits among children, adults, and older adults, to examine whether higher activity-weighted ozone–heat exposure is associated with measurable respiratory health burdens. Because the XGBoost model showed limited predictive performance, the SHAP results should be regarded as exploratory and hypothesis-generating rather than confirmatory evidence of environmental drivers.

Finally, the proposed COHER framework involves several methodological choices, including min–max normalization, multiplicative index construction, the use of an AOI-level LST, centroid-based O_3_ assignment, and 500 m buffers for surrounding environmental variables. These choices were made to maintain interpretability and consistency in a place-based screening framework, but they also introduce important limitations. The multiplicative structure highlights spaces where a high visitation frequency, an elevated O_3_ background, and higher surface thermal conditions co-occur, but it does not imply that these three components have identical physiological effects or equal epidemiological importance.

In addition, COHER should be interpreted as a relative spatial screening index rather than a clinically validated health risk metric. It does not incorporate individual dwell times, exercise intensities, age-specific vulnerability, pre-existing diseases, or exposure–response relationships. Alternative formulations, such as additive indices, weighted indices, or evidence-based health risk functions, may produce different rankings of priority spaces [57]. Future work should conduct systematic sensitivity analyses using different buffer sizes, normalization methods, weighting schemes, and exposure formulations. Such analyses would help to assess the robustness of high-risk space identification and support the development of more transferable frameworks for exposure-sensitive outdoor exercise space planning.

## 5. Conclusions

This study developed an activity-weighted framework for assessing the compound ozone–heat exposure risk in urban outdoor exercise spaces. By integrating the mobile phone signaling-derived visitation frequency, ground-level O_3_ estimates, the Landsat-derived LST, and surrounding environmental variables, the framework links environmental stressors with actual activity intensities and provides a place-based perspective for identifying outdoor exercise spaces where intensive human use coincides with unfavorable atmospheric and thermal conditions.

The results showed that the average daily visitation frequency, O_3_ exposure background, and LST presented different spatial patterns across outdoor exercise spaces. This mismatch indicates that single-factor assessments based only on pollutant concentrations, thermal conditions, or facility use may fail to identify priority spaces where the compound exposure risk is most relevant. The proposed COHER index addressed this limitation by integrating the three components into a relative activity-weighted risk indicator. The COHER values were strongly right-skewed, and the top 10% high-COHER spaces had significantly higher visitation frequencies and mean LSTs than the remaining spaces, whereas their O_3_ backgrounds did not differ significantly. This finding suggests that a high compound exposure risk is mainly associated with the spatial overlap between active population use and elevated local surface thermal conditions under broadly comparable regional O_3_ backgrounds.

The environmental comparison further showed that higher COHER was associated with significantly higher imperviousness and a more intensive surrounding built form, including higher BCR, BVD, FAR, and FAI. However, not all land cover, road network, or building morphology indicators differed significantly between the two groups. While the XGBoost model based only on surrounding environmental variables had limited predictive performance, the SHAP analysis provided exploratory evidence of relative environmental associations rather than causal effects or strong predictive controls.

Overall, this study demonstrates the value of incorporating the human activity intensity into compound environmental exposure assessment. The COHER framework can help urban managers to move beyond supply-oriented evaluations of outdoor exercise spaces and identify locations where environmental improvement may have greater exposure reduction relevance. Future planning and management should consider not only the accessibility and service coverage of exercise spaces, but also their air pollution backgrounds, thermal environments, surrounding built forms, ventilation potential, and actual intensity of use. Such an exposure-sensitive perspective can support more targeted interventions, including heat mitigation, green–blue infrastructure enhancement, ventilation-sensitive design, and time-sensitive risk communication during high-O_3_ or high-temperature periods.

## Figures and Tables

**Table 1:** Characteristics of top 10% high-COHER spaces.

Variable	All Spaces	Top 10% High-COHER Spaces	Other Spaces	*p* Value
Number of sites	490	49	441	-
DailyVisit	724.91	2764.21	498.35	<0.001
O_3_ (μg m^−3^)	82.66	82.84	82.64	0.704
LST (°C)	18.69	19.55	18.59	<0.001
COHER	0.0143	0.0618	0.0090	<0.001

Note: Values are group means unless otherwise stated. *p* values were calculated using the Mann–Whitney U test for comparisons between the top 10% high-COHER spaces and the remaining spaces. The *p* value for COHER is reported descriptively because the high-risk group was defined using the top 10% COHER threshold. O_3_, ozone concentration; LST, land surface temperature; COHER, compound ozone–heat exposure risk index.

## Data Availability

The original contributions presented in this study are included in the article. Further inquiries can be directed to the corresponding author.

## Supplementary Materials

The following supporting information can be downloaded at: https://www.mdpi.com/article/10.3390/toxics14070581/s1, Methods S1: Variable construction and extraction procedures.
