# Supplementary material for: Activity-Weighted Assessment and Environmental Drivers of Compound Ozone–Heat Exposure Risk in Urban Outdoor Exercise Spaces
Source: Toxics. 2026 Jun 30;14(7):581. doi: 10.3390/toxics14070581 (PMC13419135; doi:10.3390/toxics14070581)
Supplement: Supplementary file 1 [file toxics-14-00581-s001.zip › toxics-4370654-supplementary.pdf]

Supplementary Methods S1. Variable construction and extraction procedures

All datasets were integrated using the outdoor exercise-space AOI layer as the base spatial unit. The AOI identifier was used to join mobile phone signaling-derived visitation frequency and all extracted environmental variables. For raster-based variables, O_3 was assigned using centroid-to-grid extraction, LST was extracted as the mean value of valid pixels within each AOI, and land-cover composition was calculated within the 500-m AOI buffer. For vector-based variables, road segments and building footprints were spatially intersected with each AOI buffer, and the clipped geometries were used to calculate road-network and building morphology indicators. The resulting variables were joined into one AOI-level analytical table for index construction and statistical analysis.

S1.1. Mobile phone signaling-derived visitation frequency

Mobile phone signaling records were aggregated at the outdoor exercise-space AOI level to derive visitation frequency. For each AOI, the total number of anonymized mobile device occurrences during the study period was divided by the number of valid observation days to calculate the average daily visitation frequency, hereafter referred to as DailyVisit. DailyVisit was used as a proxy for place-based activity intensity and population exposure potential in outdoor exercise spaces. The resulting AOI-level DailyVisit values were subsequently normalized and incorporated into the construction of the activity-weighted compound ozone–heat exposure risk index.

S1.2. O_3 exposure assignment

Daily ChinaHighO_3 D1K products from 1 March to 31 March 2025 were first aggregated to generate a monthly mean O_3 surface. Specifically, the daily MDA8 O_3 values were averaged pixel by pixel:

$$O_{3,i}^{month} = \frac{1}{D}\sum_{d=1}^{D} O_{3,i,d} \quad \text{(S1)}$$

where $O_{3,i}^{month}$ denotes the monthly mean O_3 concentration of grid cell i, $O_{3,i,d}$ is the daily MDA8 O_3 concentration on day d, and D is the number of days in the study month. The resulting monthly O_3 surface represents the average of daily MDA8 O_3 concentrations during the study month.

Considering that most outdoor exercise-space AOIs were smaller than the 1-km O_3 grid cell, O_3 values were not interpreted as site-specific intra-AOI concentrations. Instead, the O_3 value of the monthly mean grid cell containing the centroid of each AOI was assigned to the corresponding outdoor exercise space. This centroid-based assignment was used to represent the regional near-surface ozone exposure background of each exercise space and to maintain a consistent extraction strategy across AOIs. The extracted O_3 values were subsequently integrated with visitation frequency and LST metrics to construct the activity-weighted ozone–heat exposure risk index.

S1.3. LST extraction

The 30 m clear-sky monthly median LST composite was used to characterize the local surface thermal environment of outdoor exercise spaces. For each outdoor exercise-space AOI, the mean LST within the AOI was extracted and used to represent the average surface thermal condition of the corresponding exercise space:

$$LST_i = \frac{1}{N_i}\sum_{j=1}^{N_i} LS\,T_{i,j} \quad \text{(S2)}$$

where LST_i denotes the mean LST of outdoor exercise space i, $LST_{i,j}$ is the LST value of pixel j within AOI i, and N_i is the number of valid LST pixels within the AOI. The extracted AOI-level LST values were subsequently integrated with visitation frequency

and O_3 exposure background to construct the activity-weighted ozone–heat exposure risk index.

S1.4. Land-cover composition within the surrounding buffer

To characterize the surrounding land-cover context of outdoor exercise spaces, land-cover composition was extracted from the CLCD. The 500-m buffer around each outdoor exercise space was used as the spatial unit for land-cover statistics. Prior to extraction, the buffer polygons were reprojected to match the coordinate reference system of the CLCD raster. Raster cells within each buffer were extracted, and invalid or NoData pixels were excluded.

To facilitate interpretation in relation to thermal exposure and environmental buffering, the original CLCD classes were reclassified into four functional land-cover groups: vegetation, blue space, impervious surface, and other land cover. Vegetation included forest, shrubland, and grassland; blue space included water bodies and wetlands; impervious surface was retained as a separate class; and cropland, snow/ice, and barren land were grouped as other land cover.

For each outdoor exercise space, the proportion of each reclassified land-cover group was calculated as:

$$P_{i,k} = \frac{N_{i,k}}{N_i} \tag{S3}$$

where $P_{i,k}$ denotes the proportion of land-cover group k within the 500-m buffer of outdoor exercise space i, $N_{i,k}$ is the number of valid pixels belonging to group k, and N_i is the total number of valid CLCD pixels within the buffer. In addition, natural land-cover proportion was calculated as the sum of vegetation and blue-space proportions. The resulting variables, including vegetation proportion, blue-space proportion, impervious surface proportion, natural land-cover proportion, and other land-cover proportion, were used to describe the surrounding environmental context of each outdoor exercise space.

S1.5. Road-network indicators extraction

Road network data were obtained from OpenStreetMap and used to characterize the traffic-related built environment surrounding outdoor exercise spaces. All road features and exercise-space buffers were projected to a common metric coordinate system. The 500-m buffer around each outdoor exercise space was used as the spatial unit for road-network statistics.

Road segments within each buffer were identified through spatial intersection. After intersection, road lengths were recalculated based on the clipped geometries to ensure that only the portions of roads falling inside each buffer were included. For each outdoor exercise space, total road density was calculated as the total length of all road segments within the 500-m buffer divided by the buffer area:

$$RD_i = \frac{L_i/1000}{A_i/1{,}000{,}000} \tag{S4}$$

where RD_i is road density in km/km^2, L_i is the road length within the buffer in meters, and A_i is the buffer area in square meters.

In addition to total road density, major road density was calculated to represent the influence of higher-level transport infrastructure. Major roads included motorways, trunk roads, primary roads, secondary roads, and their corresponding link roads. These road-network indicators were used to describe the surrounding traffic-related urban context, which may be associated with local pollution background, heat accumulation, and outdoor activity exposure conditions.

S1.6. Building morphology indicators extraction

Building morphology indicators were derived to characterize the three-dimensional built environment surrounding outdoor exercise spaces. Building footprint polygons and floor-number attributes were used as the basic data sources. All building polygons and

exercise-space buffers were projected to a common metric coordinate system before spatial calculation. Invalid geometries and records with missing or unreasonable floor-number information were removed.

The 500-m buffer around each outdoor exercise space was used as the spatial unit for building morphology statistics. Building polygons were spatially intersected with the buffer polygons, and footprint areas were recalculated based on the clipped geometries to ensure that only the portions of buildings located within each buffer were included. Based on the processed building footprints and estimated heights, eight building morphology indicators were calculated: building density, building coverage ratio (BCR), mean building height (MBH), building height standard deviation (HSD), building volume density (BVD), floor area ratio (FAR), compactness index (CI), and frontal area index (FAI).

Building density was calculated as the number of buildings per square kilometer within each buffer. BCR was calculated as the ratio of total building footprint area to buffer area:

$$BCR_i = \frac{\sum_{j=1}^{n_i} BA_{i,j}}{A_i} \quad \text{(S5)}$$

where $BA_{i,j}$ denotes the footprint area of building j within the buffer of outdoor exercise space i, n_i is the number of buildings within the buffer, and A_i is the buffer area. MBH and HSD were calculated as the mean and standard deviation of estimated building heights, respectively, to describe the vertical scale and height heterogeneity of the surrounding built environment. BVD was calculated as the total estimated building volume divided by buffer area:

$$BVD_i = \frac{\sum_{j=1}^{n_i} BA_{i,j} \times BH_{i,j}}{A_i} \quad \text{(S6)}$$

FAR was calculated as the ratio of total estimated floor area to buffer area:

$$FAR_i = \frac{\sum_{j=1}^{n_i} BA_{i,j} \times Floor_{i,j}}{A_i} \quad \text{(S7)}$$

To characterize building footprint compactness, a compactness index was first calculated for each building as $4\pi BA/P^2$, where BA is the building footprint area and P is its perimeter. The buffer-level CI was then calculated as the footprint-area-weighted mean of building-level compactness values:

$$CI_i = \frac{\sum_{j=1}^{n_i} CI_{i,j} \times BA_{i,j}}{\sum_{j=1}^{n_i} BA_{i,j}} \quad \text{(S8)}$$

FAI was used to characterize the potential obstruction of buildings to airflow. For each building, frontal area was estimated as the product of building height and the projected width of the building footprint perpendicular to a given wind direction. Because no single dominant wind direction was assumed, FAI was calculated as the average value across eight directions:

$$FAI_i = \frac{1}{M} \sum_{m=1}^{M} \frac{\sum_{j=1}^{n_i} BH_{i,j} \times W_{i,j,m}}{A_i} \quad \text{(S9)}$$

where $W_{i,j,m}$ is the projected frontal width of building j under direction m, and M is the number of directions. Higher FAI values indicate stronger potential airflow blockage by surrounding buildings.

Together, these indicators describe building density, surface coverage, vertical structure, development intensity, footprint compactness, and aerodynamic obstruction characteristics around outdoor exercise spaces. They were used to examine how surrounding urban morphology may be associated with ventilation conditions, heat accumulation, and spatial variations in compound ozone–heat exposure risk.
